# The Nonlinear Relationship Between the Aggregate Index of Systemic Inflammation and Serum Neurofilament Light Chain Levels in U.S. Adults: A Cross‐Sectional Study

**DOI:** 10.1155/mi/6698474

**Published:** 2026-04-15

**Authors:** Jun Wei, Yang Liu, Jun Zhang

**Affiliations:** ^1^ School of Basic Medical Sciences, Jilin Medical University, Jilin, 132013, Jilin Province, China, jlu.edu.cn; ^2^ Edinburgh Medical School: Biomedical Sciences, College of Medicine and Veterinary Medicine, The University of Edinburgh, Edinburgh, EH8 9YL, UK, ed.ac.uk; ^3^ Zhejiang University–University of Edinburgh Institute, Zhejiang University School of Medicine, Haining, 314400, Zhejiang Province, China, zju.edu.cn

**Keywords:** aggregate index of systemic inflammation, cross-sectional study, neurological function, NHANES, serum neurofilament light chain

## Abstract

**Background:**

Systemic inflammation is linked to chronic diseases. This study examines the relationship between the aggregate index of systemic inflammation (AISI) and serum neurofilament light chain (sNfL), a marker of neuronal damage.

**Methods:**

Data were obtained from the 2013–2014 National Health and Nutrition Examination Survey (NHANES). Participants with complete data on AISI and sNfL were included. Multiple linear regression and subgroup analyses were used to assess the association, and the nonlinear relationship was explored through smoothed curve fitting and threshold effect analyses.

**Results:**

Among 2,061 adults in NHANES 2013–2014, ln AISI was positively associated with sNfL (pg/mL) (fully adjusted β = 2.78 per 1‐unit increase; 95% CI: 1.41–4.15; *p* < 0.0001). By quartiles of ln AISI, sNfL was higher in Q4 vs. Q1 (β = 6.22, 95% CI: 3.57–8.88; *p* < 0.0001), whereas Q2–Q3 were not significant after full adjustment; *p* for trend < 0.0001. Nonlinearity analysis identified an inflection at ln AISI = 4.34: below this value the estimate was negative and nonsignificant (β = −8.82; 95% CI: −18.10–0.46; *p* = 0.0627), whereas above it the association was positive and significant (β = 3.55; 95% CI: 2.05–5.04; *p* < 0.0001; likelihood ratio *p* = 0.013). Effect modification was observed by age (*p* for interaction = 0.0068), BMI (*p* for interaction = 0.0174), diabetes (*p* for interaction = 0.0004), hypertension (*p* for interaction = 0.0436), alcohol use (*p* for interaction = 0.0133), and stroke history (*p* for interaction = 0.0033).

**Conclusion:**

In the U.S. population, higher ln AISI values were independently associated with increased sNfL levels. A nonlinear relationship was observed between ln AISI and sNfL. This suggests that AISI may represent an important marker associated with sNfL levels beyond traditional clinical factors, and further studies are needed to better explore their association and potential underlying biological mechanisms.

## 1. Introduction

The neurofilament light chain (NfL) plays a crucial role in the structural integrity of neurons, facilitating the radial extension of axons while maintaining their dimensions, morphology, and caliber [1]. In instances of neuronal injury triggered by various factors, NfL is released from damaged axons into the interstitial fluid and subsequently diffuses into both the cerebrospinal fluid (CSF) and the bloodstream. This process has been demonstrated in several neurological conditions, including ischemic stroke, in which a significant increase in NfL concentrations is observed in both CSF and the serum. Consistent correlations between CSF and blood NfL levels have been reported, supporting the use of circulating NfL as a peripheral indicator of neuronal injury [2–4]. The process of obtaining peripheral blood samples is generally simpler; therefore, measuring serum levels is considered a more practical and repeatable approach for assessing NfL quantities [3, 4]. Currently, sNfL is widely recognized as a promising indicator for a range of neurological disorders [5]. Recent studies suggest that NfL may act as a predictor for disease activity, severity, prognosis, and treatment response monitoring in multiple sclerosis (MS) [6]. One particular study found that patients with Parkinson’s disease (PD) who exhibit elevated levels of plasma NfL are at a higher risk for cognitive decline. Their results established that plasma NfL is a reliable prognostic marker for PD and can forecast clinical shifts towards mild cognitive impairment or dementia [7]. In addition to PD, NfL has been recognized as an indicator for various other neurological disorders, including stroke and Alzheimer’s disease (AD), among others [8–10].

The aggregate index of systemic inflammation (AISI), derived from routine complete blood count (CBC) parameters, incorporates neutrophil, monocyte, platelet, and lymphocyte counts [11, 12]. As a composite index derived from multiple circulating immune cell counts, the AISI reflects systemic inflammatory status [13]. Previous studies have reported associations between composite inflammatory indices such as the systemic immune‐inflammation index (SII) [14] and the systemic inflammation response index (SIRI) [15] and serum neurofilament light chain (sNfL) levels. However, whether AISI is associated with sNfL in the general population has not yet been investigated.

Therefore, we conducted a cross‐sectional analysis using data from the NHANES 2013–2014 to examine the association between AISI and sNfL levels in U.S. adults. We hypothesized that higher AISI levels would be associated with higher circulating sNfL concentrations.

## 2. Materials and Methods

### 2.1. Study Design and Data Source

**Figure 1:**
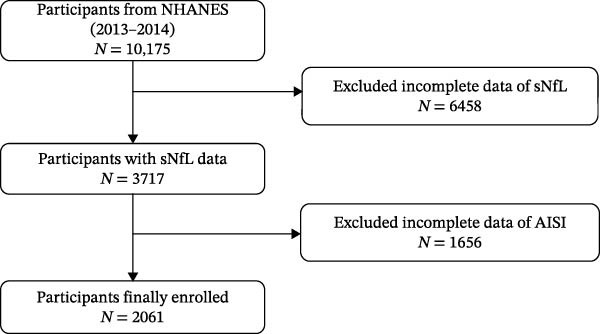
Displays the flowchart of study population.

This study employed a cross‐sectional design using data from the NHANES [16], conducted by the U.S. Centers for Disease Control and Prevention. NHANES utilizes a complex, multistage sampling method, including face‐to‐face interviews and health assessments, to provide national representative data on the health and nutritional status of the U.S. population. The analysis included participants aged 20–75 years from the NHANES 2013–2014 sample. Participants with missing data on inflammatory markers (such as platelet count, neutrophil count, monocyte count, lymphocyte count, and other relevant covariates) were excluded, as were those with missing data on sNfL (Figure 1). Multiple imputation techniques were used to minimize bias due to the missing data.

### 2.2. Definition of Exposure Variable

The variable examined in this research was the AISI, which was calculated using standard CBC tests. Blood samples were collected by certified phlebotomists under standardized NHANES procedures and analyzed in accredited laboratories. The CBC provides information on major blood cell components, including white blood cells (such as neutrophils, lymphocytes, and monocytes) and platelets. NHANES implements rigorous quality control measures to guarantee the accuracy of these measurements, which encompass standardized laboratory methods, the use of validated instruments, and routine calibration checks. To calculate the AISI, the following formula was applied: AISI = (neutrophil count × platelet count × monocyte count)/lymphocyte count [17].

For the purpose of statistical analysis, AISI values were divided into four categories corresponding to quartiles: the first quartile (Q1), second quartile (Q2), third quartile (Q3), and fourth quartile (Q4). This classification was accomplished by arranging the AISI values from all participants in an increasing order and determining the 25th, 50th, and 75th percentiles, which created four unique groups. This methodology allowed for a more in‐depth analysis and is suitable for various data distributions. To tackle the right skewness present in the data, the AISI values were transformed using the natural logarithm ln AISI [18].

### 2.3. Definition of Outcome Variables

Sera from participants aged 20–75 years, obtained from the NHANES 2013–2014 sample [17, 19], were eligible for analysis if they included surplus or pristine samples. Measurements were conducted using a highly sensitive NfL immunoassay on an Attelica automated platform. The assay uses acridinium ester (AE) chemiluminescence with paramagnetic particles. After incubation with AE‐conjugated antibodies, capture antibodies form complexes with the NfL antigen. Unbound antibodies were removed, and chemiluminescence was triggered and measured. The assay’s lower limit of quantification (LLOQ) is 3.9 pg/mL, with imputed values assigned for results below the LLOQ. No values exceeded the upper limit of quantification (500 pg/mL) [20, 21].

CBC parameters used to calculate the AISI were measured in the Mobile Examination Center (MEC) laboratory during the examination visit in accordance with the NHANES CBC protocol. sNfL was aliquoted and stored frozen (≤−70°C) under documented NHANES QA/QC procedures until centralized batch analysis at the CDC’s National Center for Environmental Health using a validated chemiluminescent immunoassay. For additional information and detailed protocols, please visit the following links: http://wwwn.cdc.gov/Nchs/Nhanes/2013-2014/SSSNFL_H.htm and https://wwwn.cdc.gov/Nchs/Data/Nhanes/public/2013/DataFiles/CBC_H.htm.

### 2.4. Covariates

Drawing on previous studies, we incorporated confounding factors that could influence the relationship between ln AISI and sNfL. Covariate data were obtained through questionnaires, physical examinations, and laboratory tests. The following covariates were included: sex, age, race and ethnicity, education, marital status, smoking status, alcohol use, body mass index (BMI, kg/m^2^), as well as the presence of diabetes and hypertension, and the household income‐to‐poverty ratio (PIR). In the NHANES, race and ethnicity information are self‐reported, based on answers to questions regarding race and Hispanic origin. As a result, participants were grouped into three categories based on their race and ethnicity: non‐Hispanic white, non‐Hispanic black, and other (including multiracial). For individuals aged 20 and above, we used the questionnaire item, “What is the highest grade or level completed or the highest degree earned?” Responses were used to categorize educational attainment into three groups: less than high school, high school or GED, and above high school. Marital status was categorized into three groups: married or living with a partner, never married, and widowed, divorced, or separated. Participants were considered alcohol drinkers if they consumed 12 or more alcoholic beverages per year. The smoking status was defined by whether participants had smoked at least 100 cigarets in their lifetime. Hypertension, diabetes, and stroke were identified based on self‐reported responses to the questionnaire, with those answering “yes” being classified as having these conditions. During physical examinations, participants’ height and weight were measured, and BMI was then calculated and categorized into three groups: under 25, between 25 and 30, and 30 or higher. Age was divided into two categories: under 60 and 60 or older. Additionally, PIR was classified into three categories: low‐income (PIR < 1.3), middle‐income (PIR 1.3–3.5), and high‐income (PIR > 3.5). For more detailed information on these covariates, please visit: https://wwwn.cdc.gov/nchs/nhanes/continuousnhanes/default.aspx?BeginYear=2013.

### 2.5. Statistical Analysis

All statistical analyses accounted for the complex survey design of NHANES, utilizing sampling weights and adjusting for data clustering. Descriptive statistics for continuous variables included means and standard deviations, while categorical variables were expressed as weighted percentages. The models were constructed in three stages: Model 1: unadjusted; Model 2: adjusted for age, sex, and race/ethnicity; Model 3: additionally adjusted for education level, marital status, PIR, smoking status, alcohol use, BMI, and histories of stroke, hypertension, and diabetes.

Multivariable linear regression models were used to estimate β coefficients and 95% confidence intervals (CIs) for the association between ln AISI and sNfL (pg/mL), with sNfL modeled on its original scale (not log‐transformed). Because AISI was right‐skewed, it was natural‐log transformed and analyzed as the primary continuous exposure; as a secondary analysis, ln AISI was categorized into quartiles (Q1–Q4), with Q1 as the reference. Nonlinearity was evaluated using generalized additive models (GAMs) with penalized smoothing splines for ln AISI; when a nonlinear pattern was suggested, two‐piecewise linear regression was applied to identify potential threshold effects, with the inflection point determined by a likelihood‐based method and models compared using likelihood ratio tests [18, 20]. Subgroup analyses (sex, age, BMI, smoking, alcohol use, hypertension, diabetes, and stroke history) and multiplicative interactions were examined, with *p* for interaction reported.

All statistical analyses were performed using R (version 4.2) and EmpowerStats (version 5.0), with results considered statistically significant when *p* < 0.05.

## 3. Results

### 3.1. Participant Characteristics

The study sample of 2,061 participants was stratified into quartiles based on the AISI (Table 1). The sNfL levels increased significantly across AISI quartiles (*p* < 0.001), with mean levels rising from 13.57 ± 10.88 pg/mL in Quartile 1–20.55 ± 32.15 pg/mL in Quartile 4. Significant demographic and clinical differences were observed across quartiles. Non‐Hispanic white individuals were more prevalent in higher AISI quartiles (*p* < 0.001). Additionally, the prevalence of hypertension and diabetes increased with higher AISI quartiles (*p* = 0.014 and *p* = 0.003, respectively). Smoking was more common in Quartile 4 (54.65%, *p* < 0.001), and higher BMI (≥30) was more frequently observed in the higher AISI quartiles (*p* < 0.001). Stroke prevalence did not differ significantly across quartiles (*p* = 0.926).

**Table 1:** Descriptive baseline characteristics of participants.

Characteristics		AISI (×10^3^/µL)^2^ quartiles				
	Total (*n* = 2,061)	Quartile 1 (*n* = 515) ≤143.11	Quartile 2 (*n* = 515) 143.11–224.40	Quartile 3 (*n* = 515) 224.40–349.49	Quartile 4 (*n* = 516) ≥349.49	*p*‐Value
Sex, *n* (%)	—	—	—	—	—	0.294
Male	988 (47.94%)	238 (46.21%)	265 (51.46%)	246 (47.77%)	239 (46.32%)	—
Female	1073 (52.06%)	277 (53.79%)	250 (48.54%)	269 (52.23%)	277 (53.68%)	—
Age (years)	—	—	—	—	—	0.457
<60	1524 (73.94%)	389 (75.53%)	368 (71.46%)	386 (74.95%)	381 (73.84%)	—
≥60	537 (26.06%)	126 (24.47%)	147 (28.54%)	129 (25.05%)	135 (26.16%)	—
Race and ethnicity, *n* (%)	—	—	—	—	—	<0.001
Non‐Hispanic white	906 (43.96%)	158 (30.68%)	238 (46.21%)	243 (47.18%)	267 (51.74%)	—
Non‐Hispanic black	369 (17.90%)	139 (26.99%)	78 (15.15%)	78 (15.15%)	74 (14.34%)	—
Other races	786 (38.14%)	218 (42.33%)	199 (38.64%)	194 (37.67%)	175 (33.91%)	—
PIR	—	—	—	—	—	0.044
<1.3	677 (35.35%)	159 (33.47%)	160 (32.92%)	167 (35.31%)	191 (39.71%)	—
1.3–3.5	631 (32.95%)	157 (33.05%)	149 (30.66%)	168 (35.52%)	157 (32.64%)	—
≥3.5	607 (31.70%)	159 (33.47%)	177 (36.42%)	138 (29.18%)	133 (27.65%)	—
Education level, *n* (%)	—	—	—	—	—	0.378
Less than high school	449 (21.82%)	114 (22.18%)	110 (21.40%)	98 (19.03%)	127 (24.66%)	—
High school or GED	428 (20.80%)	102 (19.84%)	101 (19.65%)	117 (22.72%)	108 (20.97%)	—
Above high school	1181 (57.39%)	298 (57.98%)	303 (58.95%)	300 (58.25%)	280 (54.37%)	—
Marital status, *n* (%)	—	—	—	—	—	0.005
Marriage/living with partner	1284 (62.30%)	330 (64.08%)	349 (67.77%)	311 (60.39%)	294 (56.98%)	—
Never married	394 (19.12%)	106 (20.58%)	80 (15.53%)	100 (19.42%)	108 (20.93%)	—
Divorced/widowed/separated	383 (18.58%)	79 (15.34%)	86 (16.70%)	104 (20.19%)	114 (22.09%)	—
Diabetes, *n* (%)	—	—	—	—	—	0.003
Yes	219 (10.63%)	42 (8.16%)	42 (8.16%)	61 (11.84%)	73 (14.15%)	—
No	1842 (89.37%)	473 (91.84%)	472 (91.65%)	454 (88.16%)	443 (85.85%)	—
Hypertension, *n* (%)	—	—	—	—	—	0.014
Yes	729 (35.41%)	160 (31.07%)	175 (34.05%)	185 (35.99%)	209 (40.50%)	—
No	1330 (64.59%)	355 (68.93%)	339 (65.95%)	329 (64.01%)	307 (59.50%)	—
Stroke	—	—	—	—	—	0.926
Yes	52 (2.52%)	11 (2.14%)	13 (2.52%)	14 (2.72%)	14 (2.71%)	—
No	2009 (97.48%)	504 (97.86%)	502 (97.48%)	501 (97.28%)	502 (97.29%)	—
Smoking status, *n* (%)	—	—	—	—	—	<0.001
Yes	909 (44.13%)	193 (37.55%)	206 (40.00%)	228 (44.27%)	282 (54.65%)	—
No	1151 (55.87%)	321 (62.45%)	309 (60.00%)	287 (55.73%)	234 (45.35%)	—
Alcohol use	—	—	—	—	—	<0.001
Yes	1410 (74.25%)	313 (66.74%)	355 (73.80%)	365 (77.83%)	377 (78.54%)	—
No	489 (25.75%)	156 (33.26%)	126 (26.20%)	104 (22.17%)	103 (21.46%)	—
BMI, *n* (%)	—	—	—	—	—	<0.001
<25	622 (30.42%)	187 (36.52%)	166 (32.55%)	147 (28.94%)	122 (23.69%)	—
25–30	651 (31.83%)	171 (33.40%)	170 (33.33%)	160 (31.50%)	150 (29.13%)	—
≥30	772 (37.75%)	154 (30.08%)	174 (34.12%)	201 (39.57%)	243 (47.18%)	—
Neutrophil count	4.10 ± 1.82	2.61 ± 0.78	3.50 ± 0.79	4.18 ± 0.94	6.09 ± 2.13	<0.001
Lymphocyte count	2.10 ± 0.66	2.13 ± 0.70	2.10 ± 0.63	2.09 ± 0.66	2.06 ± 0.66	0.354
Monocyte count	0.55 ± 0.18	0.41 ± 0.12	0.50 ± 0.12	0.59 ± 0.13	0.72 ± 0.19	<0.001
Platelet count	234.68 ± 59.50	203.82 ± 49.70	222.72 ± 49.01	240.55 ± 49.28	271.58 ± 66.18	<0.001
sNfL (pg/mL)	16.88 ± 20.41	13.57 ± 10.88	16.73 ± 17.38	16.68 ± 13.76	20.55 ± 32.15	<0.001

### 3.2. Association Between ln AISI and sNfL

Linear regression analysis was used to examine the association between ln AISI and sNfL (pg/mL) (Table 2). In the unadjusted model, each 1‐unit increase in ln AISI corresponded to 3.10 pg/mL higher sNfL (95% CI: 1.83–4.37; *p* < 0.0001). The association persisted after adjustment for age, sex, and race/ethnicity (β = 2.96, 1.72–4.20; *p* < 0.0001) and after further adjustment for education, marital status, poverty–income ratio, smoking, alcohol use, BMI, and histories of stroke, hypertension, and diabetes (β = 2.78, 1.41–4.15; *p* < 0.0001). When ln AISI was categorized into quartiles, sNfL was higher in Q4 vs. Q1 across all models (Model 1: β = 6.97, 4.50–9.45; Model 2: β = 6.52, 4.10–8.93; and Model 3: β = 6.22, 3.57–8.88; all *p* < 0.0001), whereas Q2 and Q3 were not statistically significant in the fully adjusted model (β = 2.46, −0.11–5.03; *p* = 0.0609; and β = 1.99, −0.65–4.62; *p* = 0.1394).

**Table 2:** Association between ln AISI and sNfL.

Exposure	Model 1		Model 2		Model 3	
	β (95%CI)	*p*‐Value	β (95%CI)	*p*‐Value	β (95%CI)	*p*‐Value
ln AISI	3.10 (1.83, 4.37)	<0.0001	2.96 (1.72, 4.20)	<0.0001	2.78 (1.41, 4.15)	<0.0001
Quartiles of ln AISI						
Q1	Reference	—	Reference	—	Reference	—
Q2	3.15 (0.68, 5.63)	0.0127	2.49 (0.08, 4.90)	0.0426	2.46 (−0.11, 5.03)	0.0609
Q3	3.11 (0.63, 5.59)	0.0140	2.50 (0.10, 4.91)	0.0414	1.99 (−0.65, 4.62)	0.1394
Q4	6.97 (4.50, 9.45)	<0.0001	6.52 (4.10, 8.93)	<0.0001	6.22 (3.57, 8.88)	<0.0001

*Note:* Model 1: unadjusted; Model 2: adjusted for age, sex, and race/ethnicity; Model 3: additionally adjusted for education level, marital status, poverty–income ratio (PIR), smoking status, alcohol use, body mass index (BMI), and histories of stroke, hypertension, and diabetes. β (pg/mL) represents the absolute difference in sNfL per 1‐unit increase in ln AISI.

**Figure 2:**
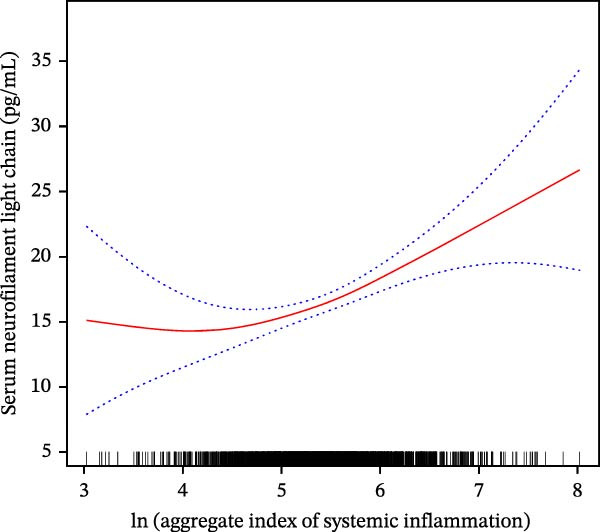
Smoothed association between ln AISI and sNfL. The red line shows the adjusted smooth fit and the dotted bands show the 95% confidence interval; tick marks on the *x*‐axis indicate the distribution of ln AISI. Models were adjusted for age, sex, and race/ethnicity; education level, marital status; PIR, smoking status, alcohol use, and BMI; and histories of stroke, hypertension, and diabetes.

Using a GAM with a penalized spline for ln AISI, we observed a nonlinear association between ln AISI and sNfL (Figure 2). The estimated curve was approximately flat at lower ln AISI and increasing at higher ln AISI, with wider confidence bands at the distributional extremes.

### 3.3. Threshold Effect Analysis

The threshold effect analysis between ln AISI and sNfL was evaluated using both a standard linear regression model and a two‐piecewise linear regression model (Table 3). The standard linear model indicated that a higher ln AISI was associated with increased sNfL levels (β = 2.78, 95% CI: 1.41–4.15, *p* < 0.0001). Below the inflection at ln AISI = 4.34, the point estimate was negative and not statistically significant (β = −8.82; 95% CI: −18.10–0.46; *p* = 0.0627), whereas above this threshold the estimated association was positive and statistically significant (β = 3.55; 95% CI: 2.05–5.04; *p* < 0.0001). The log‐likelihood ratio test (*p* = 0.013) supported the two‐piecewise model, suggesting a significant nonlinear relationship between ln AISI and sNfL.

**Table 3:** Threshold effect analysis.

Serum neurofilament light chain	Adjusted β (95%CI)
	*p*‐value
Total	—
Fitting by the standard linear model	2.78 (1.41, 4.15) < 0.0001
Fitting by the two‐piecewise linear model	—
Inflection point	4.34
In AISI < 4.34	−8.82 (−18.10, 0.46) 0.0627
In AISI > 4.34	3.55 (2.05, 5.04) < 0.0001
Log‐likelihood ratio test	0.013

*Note:* Two‐piecewise linear regression was used to assess the threshold effect of ln AISI on sNfL. A log‐likelihood ratio test with *p* < 0.05 indicates that the two‐piecewise linear model fits better than the single‐line linear model. Models were adjusted for age, sex, and race/ethnicity; educational level, marital status; PIR, smoking status, alcohol use, and BMI; and histories of stroke, hypertension, and diabetes.

### 3.4. Subgroup Analyses

Subgroup analyses were performed to examine the potential influence of demographic and health‐related factors on the relationship between the ln AISI and sNfL (Table 4). Significant interactions were observed with age, BMI, diabetes, hypertension, alcohol use, and stroke history. Specifically, age had a notable effect on the relationship (*p* for interaction = 0.0068), with individuals aged 60 years and older showing a stronger association between AISI and sNfL (β = 5.71, 95% CI: 3.16–8.25, *p* < 0.0001) compared to those younger than 60 years (β = 1.67, 95% CI: 0.10–3.25, *p* = 0.0378). BMI also significantly influenced the association (*p* for interaction = 0.0174), with a stronger relationship observed in individuals with a BMI greater than 30 (β = 4.97, 95% CI: 2.74–7.19, *p* < 0.0001), while no significant association was found in those with a BMI less than 25 (β = 0.25, 95% CI: −2.26, 2.76, *p* = 0.8441). Diabetes further moderated the relationship (*p* for interaction = 0.0004), with a stronger association in individuals with diabetes (β = 8.99, 95% CI: 5.12–12.85, *p* < 0.0001) compared to those without diabetes (β = 1.57, 95% CI: 0.12–3.02, *p* = 0.0336). Similarly, hypertension had a significant impact on the association (*p* for interaction = 0.0436), with a stronger relationship observed in individuals with hypertension (β = 4.45, 95% CI: 2.31–6.58, *p* < 0.0001), while the association was weaker in those without hypertension (β = 1.71, 95% CI: −0.00, 3.43, *p* = 0.0507). Alcohol use also modified the relationship (*p* for interaction = 0.0133), with nondrinkers showing a stronger association (β = 5.54, 95% CI: 2.95–8.13, *p* < 0.0001) compared to drinkers (β = 1.82, 95% CI: 0.25–3.39, *p* = 0.0232). Finally, stroke history significantly influenced the relationship (*p* for interaction = 0.0033), with individuals with a history of stroke showing a much stronger association (β = 16.54, 95% CI: 7.18–25.90, *p* = 0.0005), while those without a stroke history had a weaker relationship (β = 2.53, 95% CI: 1.17–3.88, *p* = 0.0030). These findings highlight the heterogeneity of the relationship between AISI and sNfL across various demographic and health‐related factors.

**Table 4:** Subgroup analyses.

Characteristics	β (95%CI), *p*‐Value	*p* for interaction
Sex, *n* (%)	—	0.2805
Male	2.04 (0.13, 3.96) 0.0368	—
Female	3.47 (1.60, 5.34) 0.0003	—
Age (years)	—	0.0068
<60	1.67 (0.10, 3.25) 0.0378	—
≥60	5.71 (3.16, 8.25) < 0.0001	—
BMI, *n* (%)	—	0.0174
<25	0.25 (−2.26, 2.76) 0.8441	—
25–30	2.19 (−0.20, 4.58) 0.0722	—
≥30	4.97 (2.74, 7.19) < 0.0001	—
Diabetes, *n* (%)	—	0.0004
Yes	8.99 (5.12, 12.85) < 0.0001	—
No	1.57 (0.12, 3.02) 0.0336	—
Hypertension, *n* (%)	—	0.0436
Yes	4.45 (2.31, 6.58) < 0.0001	—
No	1.71 (−0.00, 3.43) 0.0507	—
Smoking status, *n* (%)	—	0.6338
Yes	2.43 (0.50, 4.36) 0.0136	—
No	3.07 (1.18, 4.95) 0.0015	—
Alcohol use	—	0.0133
Yes	1.82 (0.25, 3.39) 0.0232	—
No	5.54 (2.95, 8.13) < 0.0001	—
Stroke	—	0.0033
Yes	16.54 (7.18, 25.90) 0.0005	—
No	2.53 (1.17, 3.88) 0.0003	—

*Note:* Models were adjusted for age, sex, and race/ethnicity; education level, marital status; PIR, smoking status, alcohol use, and BMI; and histories of stroke, hypertension, and diabetes.

## 4. Discussion

This study demonstrates a significant association between ln AISI and sNfL levels in a population‐based sample of U.S. adults from NHANES 2013–2014. The findings show that higher ln AISI quartiles correlate with elevated sNfL levels, with the strongest association observed in the highest quartile. Smoothed curve fitting supported a nonlinear association between ln AISI and sNfL. Subgroup analyses further highlighted that demographic and health factors such as age, BMI, diabetes, hypertension, stroke history, and alcohol use modify this relationship, underscoring the heterogeneity of the association. Notably, below the inflection point (ln AISI = 4.34), the negative estimate did not reach statistical significance and had a wide confidence interval; therefore, this finding requires confirmation in future longitudinal studies. Together, these findings suggest that the systemic inflammatory status, as reflected by AISI, is associated with variations in sNfL levels at the population level. This extends previous observations on composite inflammatory indices by demonstrating that AISI is also related to sNfL in a nationally representative sample.

Recent studies have increasingly recognized the significant role of inflammation in various health conditions. In particular, systemic inflammation markers, such as the AISI, have been associated with a range of diseases in the U.S. population, as demonstrated by data from the NHANES. For example, AISI has been linked to conditions such as erectile dysfunction [21], rheumatoid arthritis [22], chronic kidney disease (CKD) [12], and osteoporosis [23]. These findings emphasize the association between inflammation markers and disease risk and severity. The study indicates that the AISI, as a novel inflammatory marker, is significantly associated with the increased risk of cardiovascular mortality in patients with hypertension [23]. Furthermore, studies have identified a U‐shaped relationship between AISI and depression, suggesting that both low and high levels of systemic inflammation may be linked to a higher risk of depression [24]. Additionally, inflammation indices like AISI have been shown to play a role in all‐cause and cardiovascular mortality among postmenopausal women with osteoporosis or osteopenia [25].

The association between AISI and sNfL levels in our study is consistent with a growing body of evidence suggesting that systemic inflammation plays a pivotal role in neuroinflammation. The influence of systemic inflammation on the integrity and functionality of the blood–brain barrier (BBB) and neuronal health is well‐documented [26]. Elevated inflammatory markers, such as cytokines and CRP, have been shown to significantly impact neuronal structures and may contribute to the promotion of neuroinflammation [27, 28]. Specifically, pro‐inflammatory cytokines like interleukin (IL)‐6 and tumor necrosis factor‐alpha (TNF‐α) can activate immune cells and disrupt the BBB, enabling these inflammatory mediators to penetrate the central nervous system (CNS) [29]. This disruption is known to lead to neuronal damage and exacerbate neurological conditions, including AD and other neurodegenerative disorders [30].

Studies have further demonstrated that systemic inflammation alters BBB permeability by modulating the expression of tight junction proteins and increasing the influx of immune cells into the brain [31]. Elevated circulating cytokine levels can lead to morphological and transcriptional changes in brain endothelial cells, thereby compromising the BBB’s integrity [32]. Additionally, inflammation associated with conditions such as heart failure has been linked to increased BBB permeability, which facilitates neuroinflammatory processes [33].

In terms of effect modification, our subgroup analyses showed that the positive association between ln AISI and sNfL was stronger in participants with diabetes, higher BMI, and hypertension, and a modifying effect was also observed for alcohol use. These patterns align with evidence that cardiometabolic dysregulation and obesity are accompanied by systemic inflammation and increased BBB vulnerability, and that alcohol can activate neuroimmune and oxidative pathways [34]. Although stroke history was approximately evenly distributed across ln AISI quartiles, stroke emerged as the strongest modifier of the ln AISI–sNfL relationship, which suggests heightened susceptibility after stroke, potentially related to persistent BBB fragility and permeability, rather than confounding by the AISI distribution [35]. Regarding nonlinearity and the threshold finding, the two‐piecewise linear model identified an inflection at ln AISI = 4.34; below this point, the estimate was negative but not statistically significant with a wide confidence interval, whereas above the inflection, the association was positive and significant. Taken together, these results support a nonlinear association between ln AISI and sNfL and merit confirmation in longitudinal studies; given that sNfL is a sensitive but nonspecific marker of neuroaxonal injury, our conclusions are framed as associations rather than causal claims [36].

One of the key strengths of this study is its use of a large, nationally representative dataset (NHANES 2013–2014), which provides robust, generalizable findings. The inclusion of various inflammatory markers in AISI allows for a more comprehensive understanding of systemic inflammation compared to that obtained from studies using single biomarkers. Additionally, the use of sNfL as an outcome is a significant strength, as it is a reliable and widely accepted marker of neurodegeneration. Because sNfL is a nonspecific biomarker, observed associations between ln AISI and sNfL should be interpreted as exploratory and may be influenced by underlying conditions; causal or diagnostic inferences cannot be drawn from this cross‐sectional design. As such, while we can identify associations, we cannot draw conclusions about the directionality or underlying causes of the relationship. The reliance on self‐reported data for certain covariates, such as stroke history and alcohol use, may also introduce a recall bias. Furthermore, while we adjusted for a wide range of potential confounders, residual confounding is still a possibility. Per‐sample storage duration for sNfL is not available in NHANES, which precluded the formal assessment of storage‐time effects; however, specimens were handled and analyzed under standardized QA/QC procedures.

## 5. Conclusion

In the U.S. population, higher ln AISI values were independently associated with increased sNfL levels. A nonlinear relationship was observed between ln AISI and sNfL. This suggests that AISI may represent an important marker associated with sNfL levels beyond traditional clinical factors, and further studies are needed to better explore their association and potential underlying biological mechanisms.

## Author Contributions

Jun Wei collected and analyzed the data, as well as drafted the manuscript. Yang Liu and Jun Zhang revised the manuscript.

## Funding

This research was supported by the Jilin Provincial Department of Education (Grant JJKH20230542KJ) and the Jilin Provincial Department of Science and Technology (Grant YDZJ202301ZYTS455).

## Disclosure

All authors contributed to the article and approved the submitted version.

## Ethics Statement

The portions of this study involving human participants, human materials, or human data were conducted in accordance with the Declaration of Helsinki and were approved by the NCHS Ethics Review Board. The patients/participants provided their written informed consent to participate in this study.

## Consent

The authors have nothing to report.

## Conflicts of Interest

The authors declare no conflicts of interest.

## Data Availability

The survey data are publicly available on the internet for data users and researchers throughout the world (www.cdc.gov/nchs/nhanes/).
